# Global Analysis of Gene Expression Profiles in Physic Nut (*Jatropha curcas* L.) Seedlings Exposed to Salt Stress

**DOI:** 10.1371/journal.pone.0097878

**Published:** 2014-05-16

**Authors:** Lin Zhang, Chao Zhang, Pingzhi Wu, Yaping Chen, Meiru Li, Huawu Jiang, Guojiang Wu

**Affiliations:** 1 Key Laboratory of Plant Resources Conservation and Sustainable Utilization, South China Botanical Garden, Chinese Academy of Sciences, Guangzhou, P. R. China; 2 University of Chinese Academy of Sciences, Beijing, P. R. China; Ghent University, Belgium

## Abstract

**Background:**

Salt stress interferes with plant growth and production. Plants have evolved a series of molecular and morphological adaptations to cope with this abiotic stress, and overexpression of salt response genes reportedly enhances the productivity of various crops. However, little is known about the salt responsive genes in the energy plant physic nut (*Jatropha curcas* L.). Thus, excavate salt responsive genes in this plant are informative in uncovering the molecular mechanisms for the salt response in physic nut.

**Methodology/Principal Findings:**

We applied next-generation Illumina sequencing technology to analyze global gene expression profiles of physic nut plants (roots and leaves) 2 hours, 2 days and 7 days after the onset of salt stress. A total of 1,504 and 1,115 genes were significantly up and down-regulated in roots and leaves, respectively, under salt stress condition. Gene ontology (GO) analysis of physiological process revealed that, in the physic nut, many “biological processes” were affected by salt stress, particular those categories belong to “metabolic process”, such as “primary metabolism process”, “cellular metabolism process” and “macromolecule metabolism process”. The gene expression profiles indicated that the associated genes were responsible for ABA and ethylene signaling, osmotic regulation, the reactive oxygen species scavenging system and the cell structure in physic nut.

**Conclusions/Significance:**

The major regulated genes detected in this transcriptomic data were related to trehalose synthesis and cell wall structure modification in roots, while related to raffinose synthesis and reactive oxygen scavenger in leaves. The current study shows a comprehensive gene expression profile of physic nut under salt stress. The differential expression genes detected in this study allows the underling the salt responsive mechanism in physic nut with the aim of improving its salt resistance in the future.

## Introduction

Salinity is a major abiotic stress which seriously affects plant growth and crop productivity. Nearly 20% of irrigated agricultural land is under quite serious threats of salinization, and the problem of soil salinity is continuously increasing [1]. High salt concentrations impose both ionic and osmotic stresses, coupled with secondary stresses such as oxidative stress and nutritional disorders in most plants [2]. The salt overly sensitive (SOS) pathway is essential for regulating ion homeostasis in the cytoplasm and for tolerance of salt stress [2]. High concentrations of salts in the soil reduce the uptake of water by the roots. Plant responses to salinity are associated with osmotic regulation mechanisms [3]. There are several signal transduction pathways linked to high salinity, both ABA-dependent and ABA-independent. In the ABA-dependent pathway, several transcription factors (TFs) response to stress in *Arabidopsis*, including AREB/ABFs (ABA responsive element-binding factors), MYB2, MYC2, and RD26 (responsive to desiccation 26, ANAC072) [4]. In the ABA-independent pathways, DREB2s (dehydration response element-binding protein 2), NAC (NAM, ATAF1/2, CUC2) and HD-ZIP (homeodomain leucine zipper protein) are important transcription factors associated with responses to dehydration and high salinity [5]. The relationship between the ethylene signaling pathway and salt stress has also been established. TFs NAC2 and ERF4 (ethylene-responsive transcription factor 4) affect root structure and regulate other physiological pathways in *Arabidopsis* [6], [7]. Additionally, functional genes employed in carbohydrate and osmolyte metabolism, water and metal transportation, and reactive oxygen species scavenging has proved to play crucial roles in keeping osmotic homeostasis and detoxification under salt stress [8].

Transcriptomic analysis has proved to be highly valuable for analyzing plant genome expression profiles under a wide range of developmental and environmental conditions, and the results has revealed many common responses to salinity. A large number of transcriptome profiling studies on plants exposed to salt stress have been conducted such as *Arabidopsis* [9]–[11], [12], rice [13], maize [14], *Thellungiella* [15], *Populus* [16], and cotton [17]. Most of the salt-responsive genes identified are involved in transport, production of osmoprotectants, detoxification, primary energy metabolism and hormone related signal transduction processes. Using genetic engineering, a number of salt responsive genes can improve the plant salinity tolerance [18].

The physic nut (*Jatropha curcas* L.) is a small perennial tree or large shrub, which belongs to the specie of Euphorbiaceae. It is planted as potential crops for biofuel production [19]. Previous studies showed young physic nut seedlings were sensitive to high salt condition [20], but the molecular mechnism is not clear. The 100 mM NaCl can induces a moderate stress response but is not acutely lethal [21], [22]. Indeed, in our pre-experiment, we observed visible signs with the leaf chlorosis and defoliation after the treatment (data not shown). Based on the recent sequencing of its genome and the development of expressed sequence tag (EST) libraries by our group (unpublished) and other groups [23], [24], it is a valuable plant for studying the mechanisms of responses to salinity. In the study described herein, we investigated transcriptomic changes in physic nut roots and leaves exposed to 100 mM NaCl by using next-generation sequencing-based digital gene expression tag profiling. The current paper provides an overview of the transcriptome of physic nut plants exposed to salt stress, and this would be useful for understanding the molecular mechanisms underlying salt response in plants.

## Materials and Methods

### Ethics statement

No specific permits were required for the field studies described because the experimental site is owned by South China Botanical Garden, Chinese Academy of Sciences, and the Key Laboratory of Plant Resources Conservation and Sustainable Utilization undertook the management. No specific permits were required for these locations/activities, because the location is not privately-owned or protected in any way and the field studies did not involve endangered or protected species.

### Plant materials and salt treatment

Physic nut (*J. curcas* L.) cultivar GZQX0401 was used as inbred line for the genome sequencing by our research group. So we choose this cultivar as the experimental material to study its stress response. The seeds were germinated in sand and grown in trays containing a 3∶1 mixture of sand and soil in a greenhouse illuminated with natural sunlight (day/night≈14 h/10 h; daily temperature: 25∼33°C). After emergence of the first true leaf (two weeks after germination), the trays were irrigated with 1 liter of Hoagland nutrient solution (pH 6.0) once every two days at dusk. The seedlings were subjected to salt stress at the six-leaf stage (eight weeks after germination). During the stress treatment, Hoagland nutrient solution supplied with 100 mM NaCl was irrigated daily for seven days. The group irrigated with Hoagland nutrient solution was regarded as control.

Based on the change of net photosynthesis rate (Pn) of the physic nut leaves under salt stress, treated and untreated seedlings at three points were sampled. They were the early point (2 h after the start of the stress treatment); the point of rapid reduction of Pn stage (2 d after the start of the stress treatment, Pn decreased to 83% of the control), and the point of relatively stable net photosynthesis rate stage (7 d after the start of the stress treatment, Pn maintained about half of the control). Root samples comprised all root tips ca. 5–10 mm long, while leaf samples comprised blades of the third fully expanded leaf from the apex. Samples were harvested from 3 seedlings for each point, and the collection was repeated 3 times as biological replicates. Samples were frozen immediately in liquid nitrogen and stored at −80°C prior to analysis.

### RNA isolation

Total RNA of roots or leaves samples were extracted using the CTAB method [25]. The isolated RNA was subsequently treated with RNase-Free DNase I (Roche, http://www.roche.com).

### Digital gene expression library preparation and sequencing

Two biological replicates, a total of 24 samples were sequenced. These include 6 root samples and 6 leaf samples from both salt-stressed plants and control plants. Tag libraries of the samples were then prepared in parallel using an Illumina gene expression sample preparation kit and sequenced using the Illumina GAII platform at BGI-Shenzhen (http://en.genomics.cn/navigation/index.action) [26]. The raw data were submitted to the sequence read archive (SRA) at NCBI (accession number PRJNA244896). A preprocessed database of all possible CATG+17 nucleotide tag sequences was created using our genome reference database. Further information on the predicted protein-encoding genes is available at DDBJ/EMBL/GenBank under the accession AFEW00000000 (The version described in this paper is the first version, AFEW01000000). For annotation, all tags were mapped to the reference sequences including 500 bp genomic sequences behind the open reading frame, allowing no more than one nucleotide mismatch per tag. All the tags that mapped to reference sequences from multiple genes were filtered and the remaining tags were considered to be unambiguous tags. For gene expression analysis, the number of expressed tags was calculated and then normalized to TPM (number of transcripts per million tags) [27]. The sequencing saturation analysis was shown in Table S1.

Through the website IDEG6 (http://telethon.bio.unipd.it/bioinfo/IDEG6_form/index.html), the DEGs were selected within the threshold p<0.01 using Audic's algorithm with Bonferroni Correction [28], [29]. Furthermore, only the DEGs that fold change ≥1.8 were analyzed. For the gene expression pattern, genes were classified by using GeneCluster 2.0 [30] (http://www. broadinstitute.org/cancer/software/genecluster2/gc2.html). With regard to the salt responsive pathways, genes that TPM ≥5 and fold change ≥1.8 were analyzed. Biological processes enrichment of the DEGs was performed using the website of agriGO (http://bioinfo.cau.edu.cn/agriGO/index.php) [31]. The ortholog genes were identified using physic nut predicted protein sequences as query sequences to carry out Blastp searche against the *Arabidopsis* proteins through the database TAIR (http://www.arabidopsis.org/Blast/index.jsp) and NCBI (http://blast.ncbi.nlm.nih.gov/Blast.cgi). The gene best blasted with *Arabidopsis* was regarded as ortholog gene.

### Quantitative real-time PCR (QRT-PCR)

The first-strand cDNA was synthesized from 2 µg of total RNA of each sample, using M-MLV reverse transcriptase (Promega, http://www.promega.com) according to the manufacturer's instructions. QRT-PCR was performed using a Mini Option real-time PCR system (LightCycler 480) and the following program according to the manufacturer's instructions. The specific primers are listed in Table S2. *JcActin* (Genebank accession number: HM044307.1) was used as the reference gene in this experiment.

## Results

### General features of the salt stress responsive genes expression profile

Using next-generation sequencing-based digital gene expression tag profiling, we examined genome-wide changes in the transcriptomes of physic nut seedlings at 2 h, 2 d and 7 d. 17649 and 15964 protein-encoding genes with clean tags transcripts were detected in roots and leaves, respectively.

**Figure 1:**
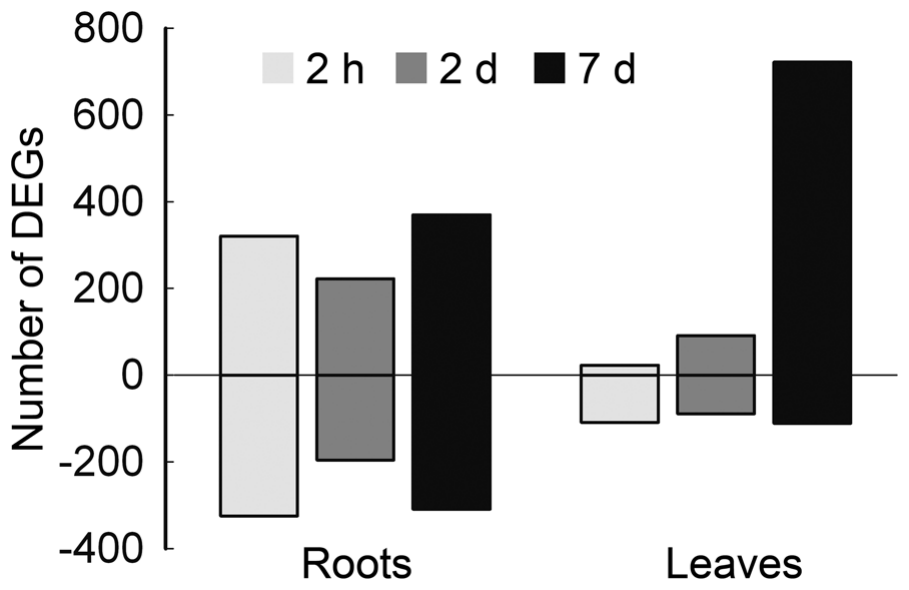
General features of the DEGs in the tested tissues. The number of upregulated genes (p value<0.01, salt/control ≥1.8) was shown in upward bars and the number of downregulated genes (p value<0.01, control/salt ≥1.8) was shown in inverted bars. DEGs, differentially expressed genes.

Total 1,504 and 1,115 DEGs associated with salt stress response in roots and leaves were determined respectively (Figure 1, Table S3). In roots, more genes were regulated at 2 h and 7 d, while there was a gradual increase in the number of upregulated genes over the time in leaves. The expression pattern of these genes in roots and leaves could be classified into six and four groups, respectively (Tables S4 and S5). In roots, the genes in cluster 0 and cluster 3 were gradually decreased during salt stress treatment, while genes in cluster 5 were gradually increased. The transcripts of cluster 2 and cluster 4 genes increased from 2 h to 2 d but decreased subsequently with different degree, which converse to cluster 1 (Table S4). In leaves, the expression pattern of genes in cluster 0 slightly upregulated at early stage but decreased at late stage. The genes in cluster 1 displayed an opposite pattern with cluster 0. The expressions of cluster 2 and cluster 3 showed peaks at 7 d were increasing gradually in different degree after the onset of salt stress (Table S5). For genes with greatest increases and reductions under salt stress at both 2 h and 2 d, or both 2 d and 7 d, data were listed in Table S6.

**Figure 2:**
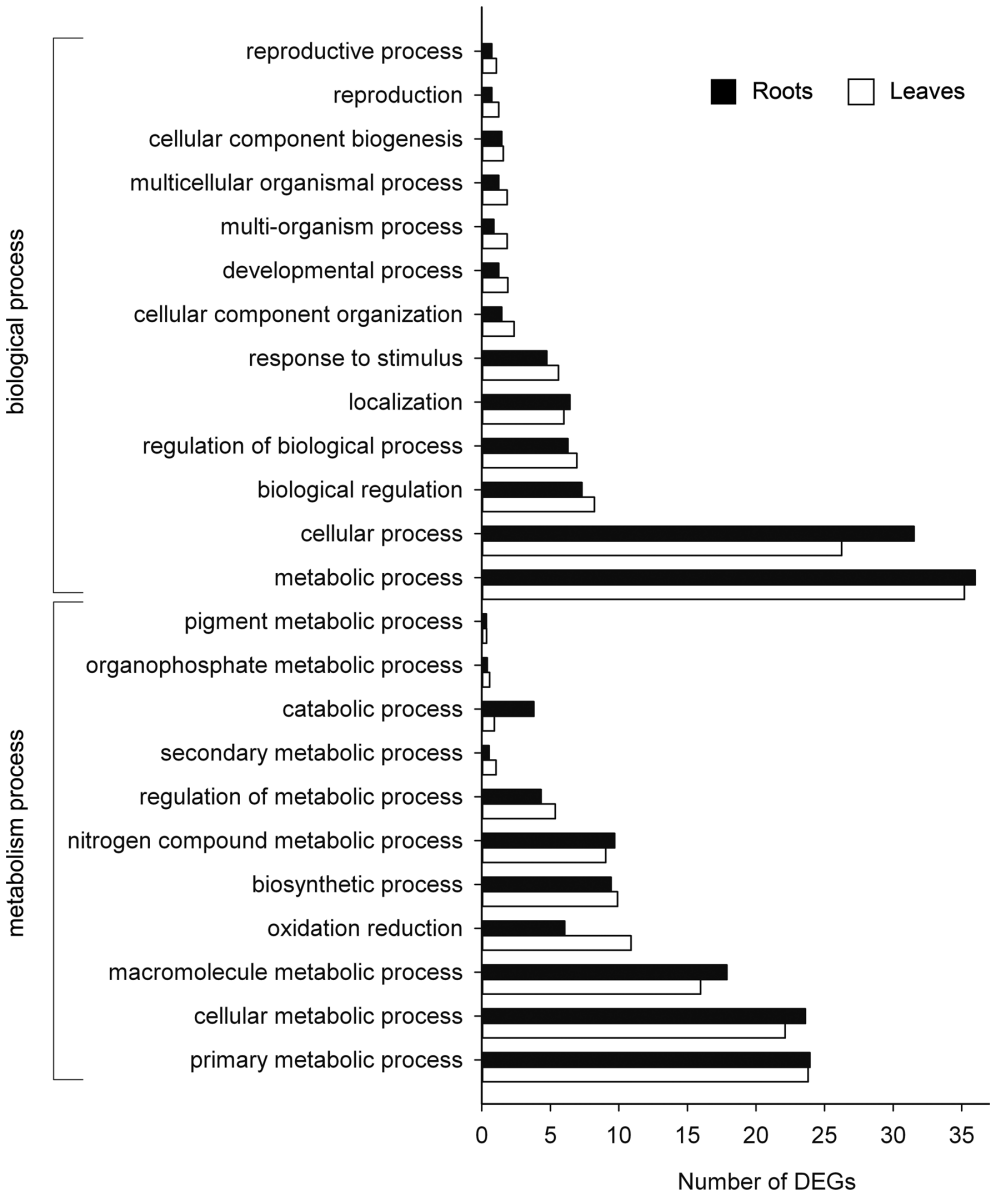
GO process analysis of the DEGs in the tested tissues.

The DEGs of roots and leaves was classified based on Gene Ontology (GO) annotation. In the biological process analysis, more genes were involved in “metabolism process”, “cellular process” and “biological regulation” (Figure 2). Specifically, in most categories of the “metabolic process”, more genes were involved in “primary metabolism process”, “cellular metabolism process” and “macromolecule metabolism process” (Figure 2).

**Figure 3:**
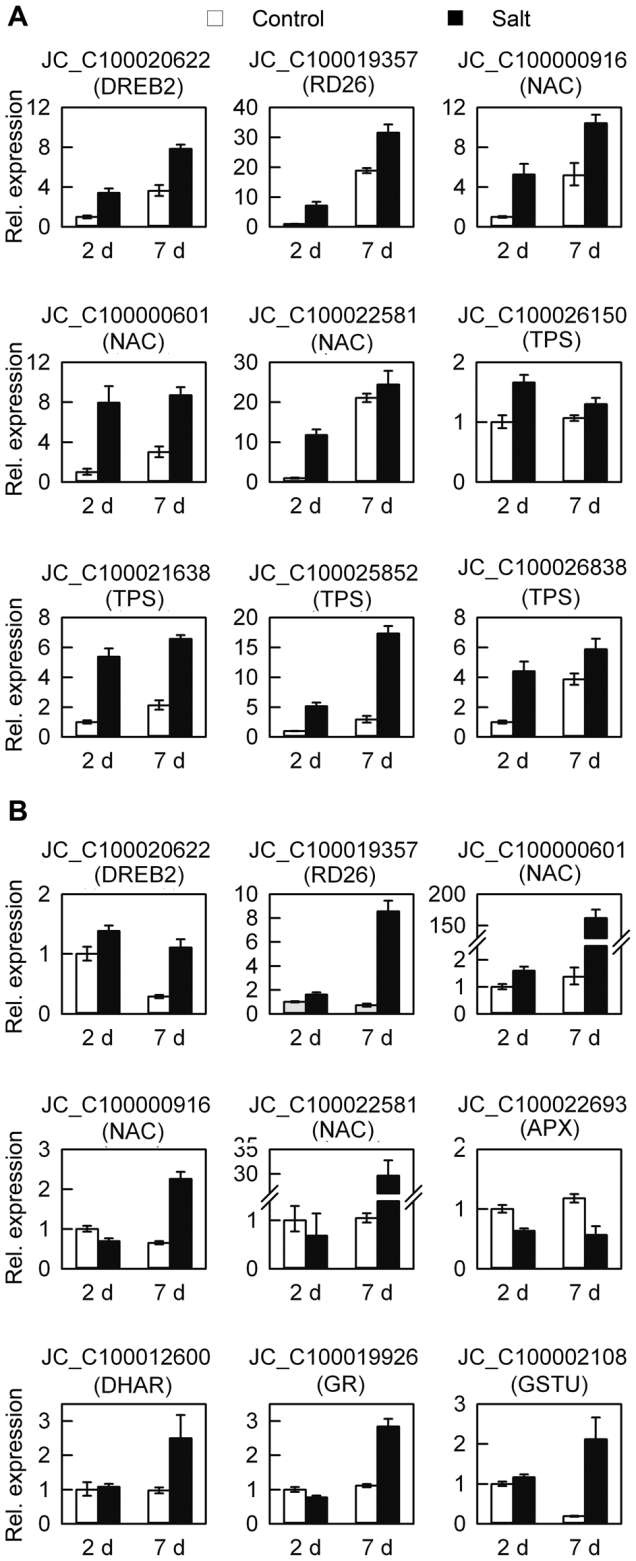
Quantitative RT-PCR analysis of selected genes under salt stress. Relative expression folds of selected genes in (A) roots and (B) leaves after the onset of salt stress. Error bars indicate the SD for three independent replicates. APX, ascorbate peroxidase; DHAR, dehydroascorbate reductase; DREB2, dehydration response element-binding protein 2; GR, glutathione-disulfide reductase; GSTU, glutathione S-transferase TAU family; NAC, NAM, ATAF1;2, CUC2; RD26, responsive to desiccation 26; TPS, trehalose-phosphatase/synthase.

To assess whether the digital expression data could be confirmed by an alternate method, fifteen genes involved in ABA signaling pathway, trehalose synthesis and reactive oxygen scavenging were selected for QRT-PCR (Figure 3). The results were generally consistent with the digital gene expression tag profiling, which confirmed the reliability of the digital expression data.

### Salt stress responsive genes in roots

Ionic homeostasis related genes. It is critical to maintain the intracellular ionic homeostasis for plants exposed to salt stress. The SOS pathway is a major way to regulate ionic homeostasis. However, the related genes of physic nut were not responsive at transcription level, except one plasma H^+^-ATPase (AT3G60330) ortholog (JC_C100008021) was upregulated at 2 h in roots and downregulated subsequently (Table S7).

**Figure 4:**
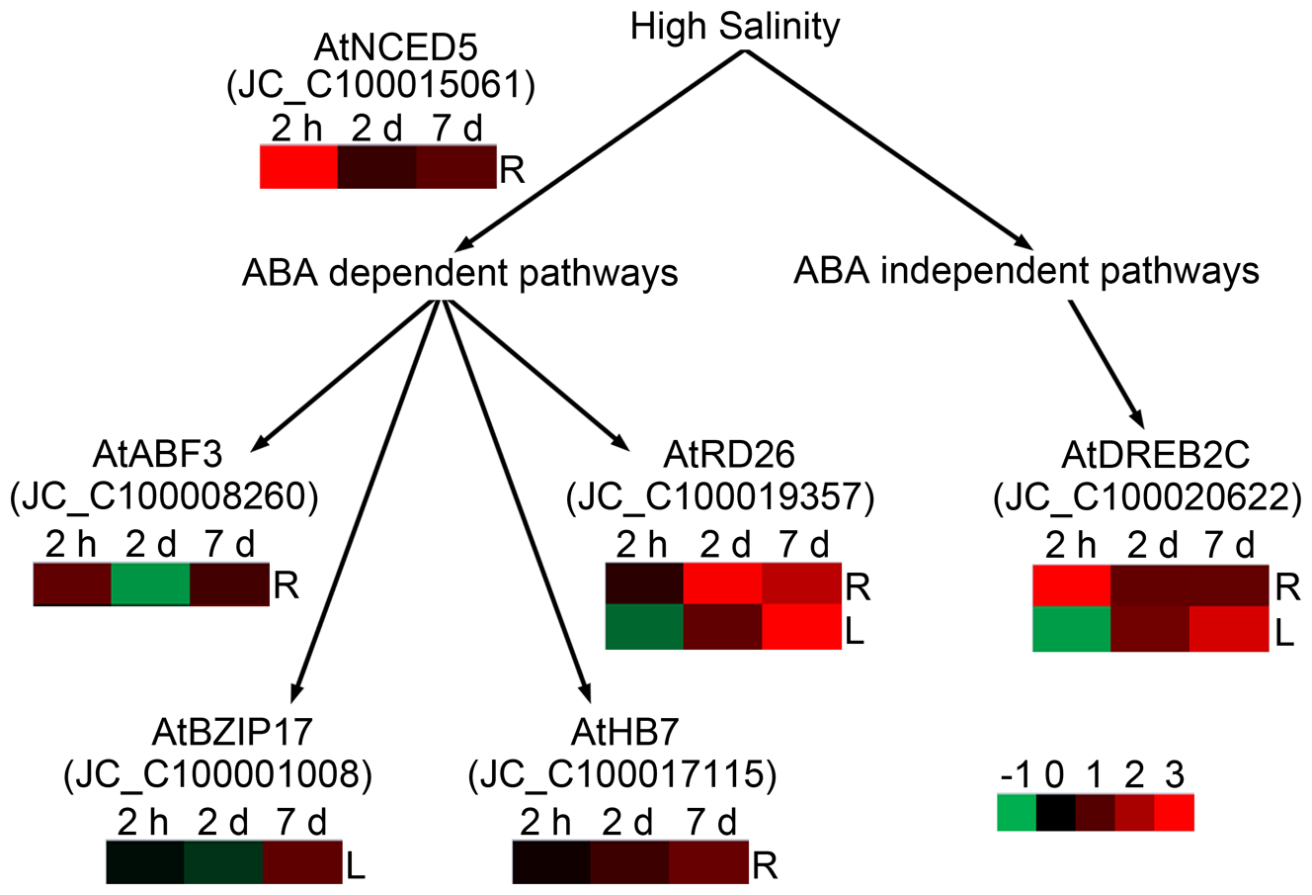
Expression profiles of osmotic signaling related genes. This figure was modified from transcriptional regulatory networks of abiotic stress signals and gene expression [32]. Genes are showed based on alignment with *Arabidopsis*. Red and green color gradients indicate an increase or decrease in transcript abundance based on log2 transformation of average change fold, respectively. ABF3, abscisic acid responsive elements-binding factor 3; BZIP, Basic-leucine zipper; DREB2, dehydration response element-binding protein 2; HB7, homeodomain leucine zipper protein 7; NCED5, 9-cis-epoxycarotenoid dioxygenase 5; RD26, responsive to desiccation 26; R, Roots; L, Leaves.

Osmotic regulation related genes. Several transcription factors are proposed components of the osmotic regulation signal transduction pathways in plants [3], [5], [32]. JC_C100020622, ortholog of *Arabidopsis* DREB2C (AT2G40340) which is a downstream gene in the ABA-independent pathway, was upregulated (Figure 4, Table S7). NCED5 (9-cis-epoxycarotenoid dioxygenase 5) (AT1G30100) ortholog (JC_C100015061), which encodes the key enzyme in ABA biosynthesis pathway, was upregulated at 2 h. One HD-ZIP gene, JC_C100017115, ortholog of ATHB7 (AT2G46680), was upregulated (Figure 4, Table S7). The ABF3 (AT4G34000) ortholog (JC_C100008260) was upregulated at 2 h, which functions in the downstream of the ABA-dependent pathway. The RD26 (AT4G27410) ortholog (JC_C100019357) was upregulated at late stage in roots. The details of other candidate genes in ABA signaling pathway response to salt stress in roots were shown in Table S7. Downstream of this transcriptional regulatory network, a number of osmolyte (such as trehalose and proline) synthesis genes are involved in osmotic homeostasis and the stabilization of proteins and cellular structures [33]. In this study, the delta-1-pyrroline-5-carboxylate synthetase P5CS1 (AT2G39800) ortholog (JC_C100020740) in proline biosynthesis was upregulated at 7 d (Table S7). In the trehalose biosynthesis pathway, four putative trehalose phosphatase/synthase genes, the ATTPS9 orthologs (JC_C100021638 and JC_C100026838), the ATTPS11 (AT2G18700) ortholog (JC_C100025852), ATTPS7 (AT1G06410) ortholog (JC_C100026150) were downregulated at 2 h but upregulated at late stage, but the ATTPS5 (AT4G17770) ortholog (JC_C100001653) was expressed in a contrary pattern (Table S7). At the later stages of maturation, the seeds of many species acquire the capacity to withstand the removal of the majority of their water, i.e. they acquire desiccation tolerance. The mechanism of desiccation tolerance is thought to involve the synthesis of protective molecules. ABA and ABA related signaling pathways play an important role in the acquisition of desiccation tolerance in seeds [34]. A number of genes are particularly highly expressed in late development stage seeds of physic nut [26]. Aquaporins are a family of integral membrane proteins that facilitate the transport of small molecules such as water, small uncharged solutes, and gases across biological membranes [35]. In roots, one plasma membrane intrinsic proteins PIP2;2 (AT2G37170) ortholog (JC_C100012358) and four tonoplast intrinsic proteins (TIP) orthologs, TIP4;1 (AT2G25810) ortholog (JC_C100025807), TIP1;1 (AT2G36830) ortholog (JC_C100021106), and TIP1;3 (AT4G0147) orthologs (JC_C100021242 and JC_C100005465) were upregulated at early stage but downregulated at late stage (Table S7).

Ethylene signaling related genes. Ethylene is involved in plant stress responses and root development [36]. The putative ACO4 (1-aminocyclopropane-1-carboxylate oxidase 4) (AT1G05010) ortholog (JC_C100009042) and S-adenosylmethionine synthetase SAM2 (AT4G01850) ortholog (JC_C100010298) associated with the ethylene biosynthesis pathway was downregulated at 2 h but upregulated at late stage (Table S7). In the ethylene signal transduction pathway, AT2G40940 ortholog (JC_C100024264) and AT3G23150 ortholog (JC_C100014744) were upregulated at 7 d in roots (Table S7). The NAC transcription factor ATNAC2 (AT3G15510) ortholog (JC_C100019647), which downstream of EIN2 in ethylene signal transduction pathway, was downregulated at 7 d (Table S7).

Apart from the plant hormone response orthologs mentioned above, a number of transcription factors including ARF (3), NAC (5), WRKY (3) and ERF (9), MYB (10) families showed different expression patterns (Table S7). The DEGs encoded protein kinases were predominate in leucine-rich repeat protein kinase and protein kinase superfamily protein (Table S7).

Cell wall related genes. Fasciclin-like arabinogalactan proteins (FLAs) have been reported to function as adhesion molecules and to be involved in secondary-wall formation in plants [37]. Chitinase not only functions as a defense enzyme against fungal and bacterial invaders, but also plays a role in altering root system architecture in response to multiple environmental conditions in various plant species [38]. Expansins are a group of cell-wall proteins required for plant cell wall extensibility and growth [39]. In this study, the expression change of these protein related genes in roots were shown in Table S7. The FLA2 (AT4G12730) ortholog (JC_C100006978) was downregulated especially at 7 d. Six chitinase genes were downregulated at 2 h and upregulated at 2 d and/or 7 d. The ATEXPA20 (AT4G38210) ortholog (JC_C100005919) and ATEXPB3 (AT4G28250) ortholog (JC_C100007573) were upregulated at 2 h but downregulated at subsequently time points. While the ATEXPA1 (AT1G69530) ortholog (JC_C100022065), ATEXLB1 (AT4G17030) (JC_C100010504) and ATEXLB1 (AT4G17030) ortholog (JC_C100008042) were responded conversely, that downregulated at 2 h but upregulated at 2 d and 7 d. In addition, ATEXPA12 (AT3G15370) ortholog (JC_C100003324) was downregulated at 2 h and 7 d but upregulated at 2 d. Furthermore, there were five putative xyloglucan endotransglucosylase/hydrolase genes upregulated at 2 h or 2 d. These results indicate that there is reconstruction of the cell wall structure of physic nut roots in response to salt stress.

### Salt stress responsive related genes in leaves

Osmotic regulation related genes. Raffinose accumulated in leaves of plants experiencing water deficit [40]. Two raffinose synthase genes, AT3G57520 ortholog (JC_C100008745) and AT5G20250 ortholog (JC_C100008342) were upregulated at 7 d in leaves (Table S8). Late Embryogenesis Abundant (LEA) protein maintaining protein structure stabilization confers plants tolerance to salt stress [41]. Three LEA protein genes (JC_C100033598, JC_C100013430 and JC_C100004237) and four heat shock protein genes in leaves were upregulated in different pattern (Table S8). After comparisons, we found that a number of genes specifically expressed in late stage of seed development were upregulated at 2 d and/or 7 d in leaves (Table S8). The expression of two aquaporin orthologs was changed. The PIP2;2 (AT2G37170) ortholog (JC_C100012358) was upregulated at 7 d but the BETA-TIP (AT1G17810) ortholog (JC_C100005178) was downregulated from 2 d (Table S8).

Oxidative stress related genes. Salt stress, like many environmental stresses, generates reactive oxygen species (ROS) in many processes. As a key enzyme in ROS scavenging, superoxide dismutase (SOD) is an endogenously produced intracellular enzyme and presents in essentially every cell in the body to eliminate intracellular superoxide radicals [42]. The Fe superoxide dismutase 3 (AT5G23310) ortholog (JC_C100011161) was upregulated in leaves after the salt stress (Table S8). The glutathione S-transferase (GST) family is composed of many isozymes involved in diverse intracellular events and has the capacity to influence response to environmental stresses [43]. In this study, the ortholog (JC_C100002108) of ATGSTU19 (AT1G78380), which encodes a member of Tau GST gene family, was upregulated at 7 d in leaves. The glutathione-ascorbate cycle is one of the most important antioxidant protection systems for removing H_2_O_2_ generated in cytosol, mitochondria, chloroplasts and peroxisomes [44], [45]. In this cycle, the APX4 (AT4G09010) ortholog (JC_C100022693) was downregulated at 7 d. The glutathione reductase gene (JC_C100019926) and the putative dehydroascorbate reductase gene (JC_C100012600), orthologs of ATGR1 (AT3G24170) and ATDHAR1 (AT1G19570) respecively, were upregulated at 7 d (Table S8). Flavonoids, function in eliminating singlet oxygen and stabilizing the chloroplast outer envelope membrane [46], [47]. In this study, three genes (JC_C100032884, JC_C100025615 and JC_C100013991) required for flavonoid biosynthesis were upregulated at 7 d (Table S8). Additionally, in the most salt responsive DEGs (fold change ≥3) at 7 d in leaves (Table S9), photosynthesis related orthologs AT3G56650 (JC_C100001009), AT5G66570 (JC_C100017660) and AT1G15820 (JC_C100002820), were significantly downregulated. The plastocyanin AT1G20340 ortholog (JC_C100031097) involved in photosynthetic electron transfer was also significantly downregulated (Table S9). Additionally, several significantly upregulated genes were involved in protein stability and degradation process (Table S9).

Ionic homeostasis and osmotic signaling related genes. Distinct from roots in maintenance of intracellular ionic homeostasis, the AT1G19910 ortholog (JC_C100012875), involved in ATP hydrolysis coupled proton transport, was upregulated at 7 d (Table S8). While the ATNHX6 (AT1G79610) ortholog (JC_C100002813), encoding a Na^+^/H^+^ antiporter, was downregulated at 2 h in leaves (Table S8). In ABA dependent pathway for osmotic and salt regulation, BZIP17 (AT2G40950) ortholog (JC_C100001008) was upregulated at 7 d, the RD26 (AT4G27410) ortholog (JC_C100019357) was upregulated at late stage in leaves (Figure 4, Table S8). In ABA independent pathway, the DREB2C (AT2G40340) ortholog (JC_C100020622) was upregulated at late stage (Figure 4, Table S8). The details of other candidate genes in ABA signaling pathway response to salt stress in leaves were shown in Table S8. Other response transcription factor genes include MYB (5), NAC (4), ERF (2), bHLH (3), AUX/IAA (2), C3H (2) (Table S8). Protein kinases were major in protein kinase superfamily protein, calcium-dependent protein kinase and leucine-rich repeat receptor-like protein kinase family protein (Table S8).

## Discussion

Using next-generation Illumina sequencing technology analysis, we successfully identified sets of upregulated and downregulated genes in physic nut plants subjected to salt stress. Gene ontology (GO) analysis revealed that the same categories of salt-responsive genes were regulated in both roots and leaves, but with different number of genes in each tissue (Figure 2). This suggests similar roles for these categories of genes in the general salt stress response. Additionally, large number of genes was involved in “metabolic process” and the most responsive one was “primary metabolic process” (Figure 2). It indicated that these processes are important for the salt stress response. Based on the pathway analysis in physic nut roots, salt response related genes major involved in osmotic regulation (through proline and trehalose synthesis, aquaporins and related signaling transduction) and cell wall remolding. However, physic nut leaves response to salt stress were predominately by osmotic regulation, antioxidative regulation and protein modification.

The SOS pathway has been fully elucidated with respect to the control of ionic homeostasis in Arabidopsis [48]. However, no significant change of genes related to SOS pathway was observed at transcription level under the present conditions in physic nut. Root plasma membrane and tonoplast H^+^-ATPases are involved in the adaptation of plants to salinity [49]. One otholog of H^+^-ATPases was significantly upregulated at 2 h in physic nut roots (Table S7), which responsible for generating the proton motive force for the antiporters, driving Na^+^ ions out of the cytosol.

It is widely accepted that short term (hours to few days) of exposure to salt stress leads to osmotic effects, while prolonged exposure can bring about ionic toxicity [50]. A number of transcription factors which function in both salt and osmotic stress tolerance in plants have been identified downstream of the ABA-dependent and -independent signaling pathways [3], [5], [32]. The upregulation of a NCED gene, which encodes a key enzyme in ABA biosynthesis, was upregulated in physic nut roots exposed to salt stress (Figure 4, Table S7). Several orthologs of Arabidopsis genes involved in the transcriptional regulatory network in the physic nut were upregulated under the salt stress, which suggested these signaling pathways were conserved in the physic nut and *Arabidopsis*. Downstream of this transcriptional regulatory network, a number of osmolyte (such as trehalose and proline) synthesis genes (Table S7) were upregulated in physic nut roots exposed to salt stress. These proteins play an important role in maintaining the osmotic balance and cellular structures in physic nut.

Aquaporins facilitate water transport across cellular membranes and are, therefore, believed to play an important role in water homeostasis [51]. Genes encoding aquaporins were differentially regulated during salt stress in physic nut plants: five genes were upregulated at 2 h and/or 2 d, but downregulated at 7 d in roots during salt stress (Table S7). Such different changes in aquaporin transcripts during salt stress have also been observed in maize [52] and *Arabidopsis* [53]. In the transcriptome data of *Arabidopsis* roots, the respective aquaporins, which located in plasma membrane or tonoplast, were downregulated after the onset of salt stress [12]. These expression patterns of aquaporin genes during salt stress may reflect roles in increased water uptake in roots, limiting initial water loss during the early stage of salt stress, and maintaining the water status of plant cells as the stress continues.

The involvement of the ethylene signaling pathway in salt stress response has been reported in plants [6], [54]. In this study, a number of genes involved in ethylene biosynthesis and the ethylene signaling pathway were downregulated at 2 h but upregulated at 2 d and/or 7 d in roots exposed to salt stress (Table S7). In *Arabidopsis*, ATNAC2 is continuously upregulated in 24 h after 200 mM NaCl treatment, and it functions downstream of the ethylene signaling pathway for the regulation of root development [6], [7]. Its ortholog in physic nut was upregulated at early stage but downregualted at 7 d in roots exposed to salt stress (Table S7). These results imply the involvement of the ethylene signaling pathway in response to salt stress in physic nut, and their transcriptional regulatory network in physic nut was conserved with *Arabidopsis*.

The plant cell wall is the extracellular matrix; it contains less than 10% of the proteins (by dry weight) of the primary wall [55]. These proteins are recognized as critical components in maintaining the physical and biological functions of the extracellular matrix. Most extracellular matrix proteins belong to large families that include enzymes, expansins, and glycoproteins [56]. FLAs, chitinases, expansions are three major glycoproteins to modify the dynamic structure of the plant cell wall to allow the plant to adapt to environmental stresses [56], [57]. FLAs are a subclass of arabinogalactan proteins (AGPs), as one of the endogenous substrates for plant endochitinases, which are important for the integrity and elasticity of the plant cell wall matrix [58], [59]. HOT2/AtCTL1, encodes a chitinase-like protein plays a role in cell wall synthesis and cell elongation, and its function is modulated by environmental stimuli that alter organ morphology [57], [60]. Mutants of *hot2* have an aberrant tolerance to salt stresses, and accumulated high levels of Na^+^ in cells under either normal or NaCl stress conditions [60]. Expansins are a class of cell wall proteins that participate in the cell wall loosening process [39]. The individual expansin proteins may perform distinct cellular functions. Ectopic expression of expansin 3 or expansin β1 causes enhanced salt stress sensitivity in *Arabidopsis* [61], however, overexpression of TaEXPB23 in tobacco confers tolerance to salt stress by enhancing water retention ability and decreasing osmotic potential [62]. Wall-loosening β-expansins have been found to be abundant in salt-resistant hybrid maize leaves and confer salt tolerance [63]. In this study, the expression level of the FLA gene was downregulated, which is consistent with that in *Arabidopsis* [37]. Eight chitinase genes and four expansin genes were upregulated at late stage in roots exposed to salt stress (Table S7). Additionally, four chitinase genes and three expansin genes of them were shown the same expression pattern with that in *Arabidopsis*, while other were not responded or showed different pattern with *Arabidopsis* [12]. It illustrated that these genes may be specific for recomposing of wall matrix glycoproteins in salt-stressed physic nut roots. This recomposing may result in the incorrect modification of polysaccharides attached to glycoproteins, which play an essential role in maintaining appropriate cell sizes and forming the fine cell-wall matrix that enhances the physical barrier against salt stress.

The physic nut leaves exhibited an effective osmotic adjustment under moderate salinity, mainly via Na^+^ and Cl^−^ accumulation to maintain their hydration status [22]. Orthologs of H^+^-ATPase and NHX were affected in physic nut leaves (Table S8). For the osmotic regulation in leaves, accumulated ABA in roots can be uploaded to shoots, where it can regulate plant development, desiccation tolerance of seeds and osmotic balance in leaves [64]. In the ABA signaling pathway, othologs of several TFs including DREB2, bZIP, and NAC were upregulated at 7 d in leaves (Table S8). Downstream of ABA signaling, two raffinose synthesis genes were upregulated in physic nut leaves exposed to salt stress (Table S8). Compared to roots, the proline and trehalose biosynthesis related genes were not response. It suggested that raffinose is the major osmoprotectant in physic nut leaves.

Exposure of plants to salt stress increases the production of reactive oxygen species (ROS) which cause oxidative damage to a range of cellular components, including membrane lipids, proteins, and nucleic acids [65]. Previous studies have reported that reactive oxygen species (ROS) scavenging plays a key role in salt-stress responses in plants [66]. As the first line of defense against oxidative damage, SODs are usually induced by salinity to dismutate O^2−^ rapidly into oxygen and H_2_O_2_, which subsequently removed through various pathways. In this study, one Fe-SOD exhibited upregulated expression in leaves exposed to salt stress (Table S8). It suggested that the upregulated SOD in leaves may involved in protection the membrane structure of chloroplast from damage under salt stress, and then maintain the balance of photosynthesis process in physic nut. The glutathione-ascorbate cycle is one of the most important antioxidant protection systems for removing H_2_O_2_ generated in cell organelles [44], [45]. The expression of genes encoding several enzymes in this cycle was upregulated in physic nut plants under salt stress (Table S8). Flavonoids have antioxidant functions in higher plants [46], [47]. In this study, several genes that take part in flavonoids biosynthesis were upregulated at 7 d after the onset of salt stress (Table S8). This result suggests that flavonoids may have antioxidant functions in physic nut under the salt stress. Salt stress affects plants photosynthesis, which is induced primarily by the osmotic effects of salt excess in roots. Previous studies showed significant reduction in net photosynthesis rate of physic nut exposed to salt stress [20], [67]–[69]. In the current study, photosynthesis related genes were significantly downregulated at 7 d in leaves (Table S9). This downregulation may closely associate with inhibition of ROS accumulation in leaves. It also proved the photoprotection mechanism that dissipation of excess photosynthetic energy contributes to salt tolerance [69].

Many highly abundant transcripts in mature stage seeds encoding LEAs and HSPs were upregulated in physic nut leaves at 7 d (Table S8). These proteins function in maintaining cell turgor and cellular redox balance, and the stabilization of proteins and cellular structures, which may enhance the salt tolerance of physic nut. Additionally, several genes involved in proteolysis and ubiquitin/proteasome-dependent proteolysis were upregulated at 7 d. These proteins are speculated to prevent the aggregation of nascent or damaged proteins [70].

Conclusively, we characterized the comprehensive transcriptome of the woody plant physic nut using the Illumina sequencing technology. The genes related to osmolyte synthesis were in different pathways. In roots, the major regulated genes were involved in trehalose synthesis, while in leaves, the major regulated genes were involved in raffinose synthesis. Moreover, a number of genes related to cell wall structure and composition changes in roots and reactive oxygen scavenger in leaves were responded. There may be other mechanisms in physic nut response to salt stress under different treatment condition, which need to be studied in future. This transcriptome data could provide significant insights into the underlying molecular mechanisms that govern the response to salt stress in physic nut, and may provide a new resource for genetic engineering of salt tolerance in physic nut or other crops.

## Supporting Information

Table S1 **Sequencing saturation analysis.**
(DOCX)

Table S2 **Primers and corresponding sequences used for quantitative RT-PCR analysis.**
(XLSX)

Table S3 **Overview of salt-responsive genes in physic nut seedlings under salt stress.** Fold change ≥1.8, p value<0.01 were counted.
(DOCX)

Table S4 **Gene expression pattern of DEGs in roots.** This table provides information on expression patterns of 1,504 genes (expression level changed over 1.8-fold in at least one time point) in physic nut roots.
(XLSX)

Table S5 **Gene expression pattern of DEGs in leaves.** This table provides information on expression patterns of 1,115 genes (expression level changed over 1.8-fold in at least one time point) in physic nut leaves.
(XLSX)

Table S6 **Detailed information of the DEGs regulated at two time points.**
(XLSX)

Table S7 **Salt responsive pathways related genes in roots.**
(XLSX)

Table S8 **Salt responsive pathways related genes in leaves.**
(XLSX)

Table S9 **Detailed information of the most up- and downregulated genes in leaves at 7 d.**
(XLSX)
