# Supplementary material for: Global Analysis of Gene Expression Profiles in Physic Nut (Jatropha curcas L.) Seedlings Exposed to Salt Stress
Source: PLoS One. 2014 May 16;9(5):e97878. doi: 10.1371/journal.pone.0097878 (PMC4023963; doi:10.1371/journal.pone.0097878)
Supplement: Table S1 — Sequencing saturation analysis. (DOCX) [file pone.0097878.s001.docx]

**Table S1. Sequencing saturation analysis.**

| Roots-1 | Control | Salt stress |
| --- | --- | --- |
| 2 h | 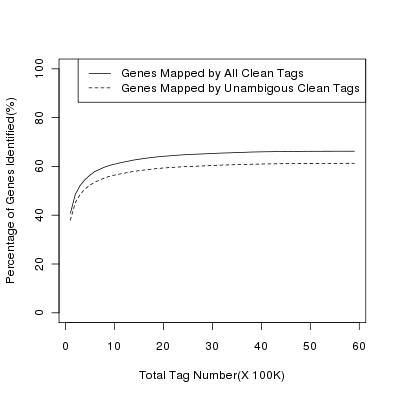 | 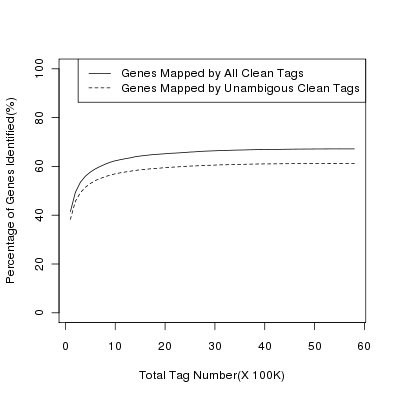 |
| 2 d | 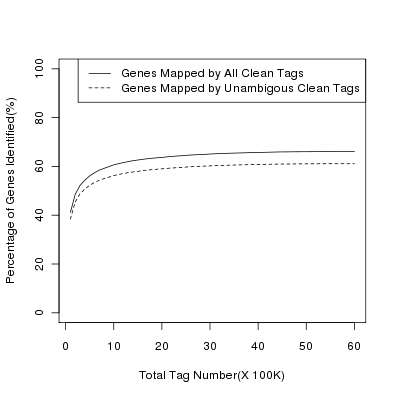 | 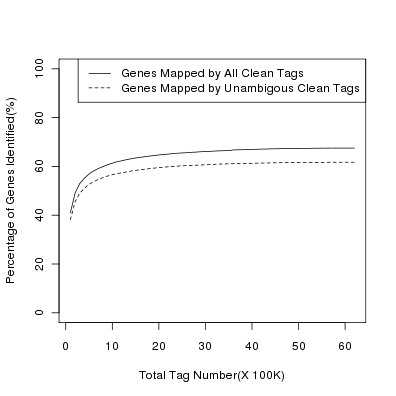 |
| 7 d | 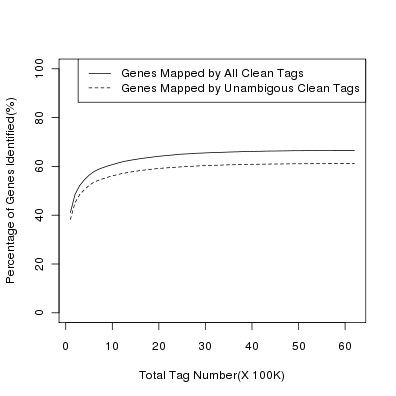 | 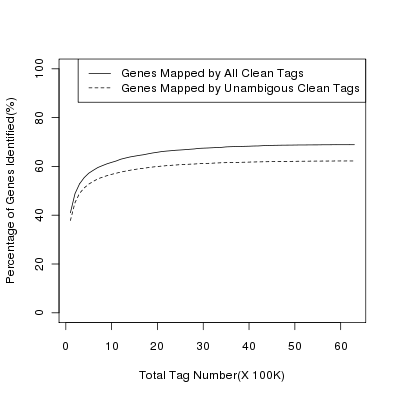 |

**Continues Table S1**

| Roots-2 | Control | Salt stress |
| --- | --- | --- |
| 2 h | 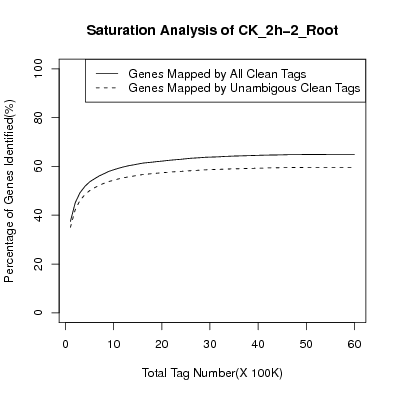 | 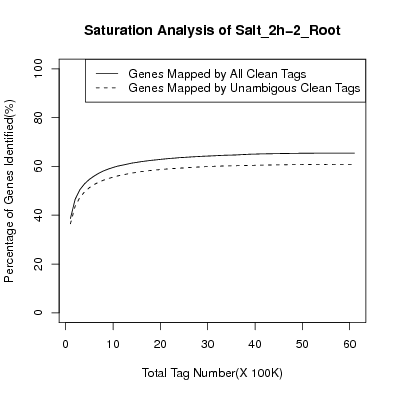 |
| 2 d | 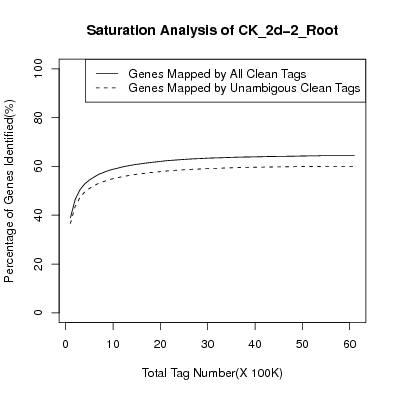 | 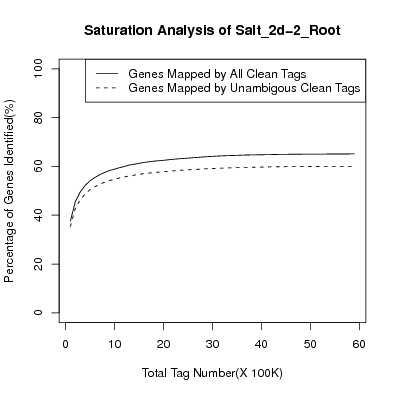 |
| 7 d | 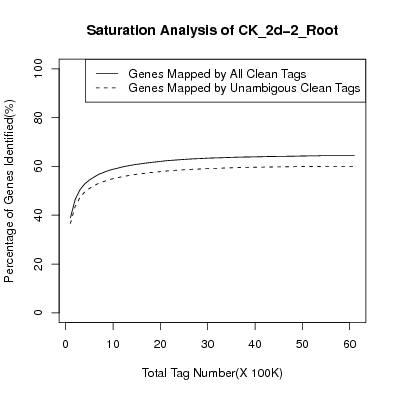 | 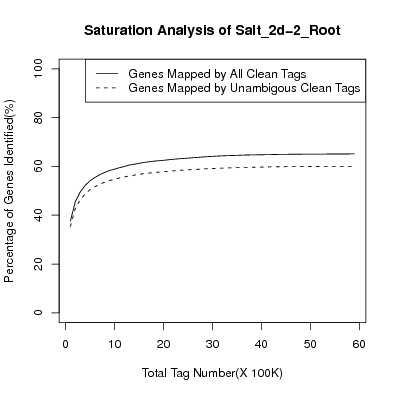 |

**Continues Table S1**

| Leaves-1 | Control | Salt stress |
| --- | --- | --- |
| 2 h | 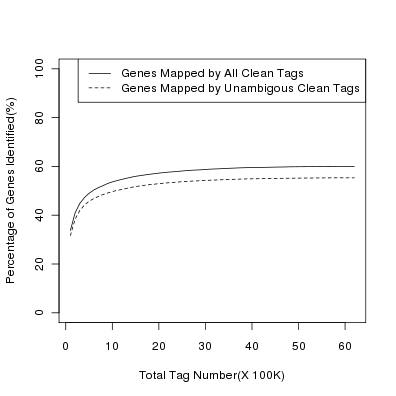 | 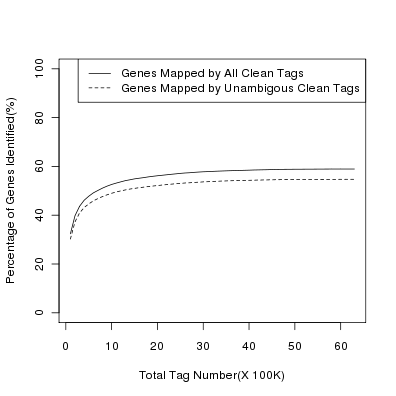 |
| 2 d | 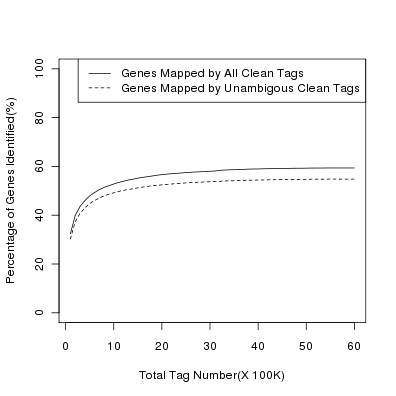 | 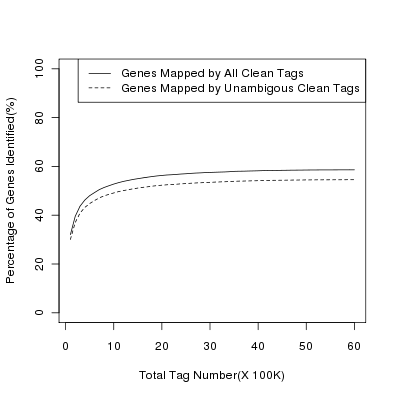 |
| 7 d | 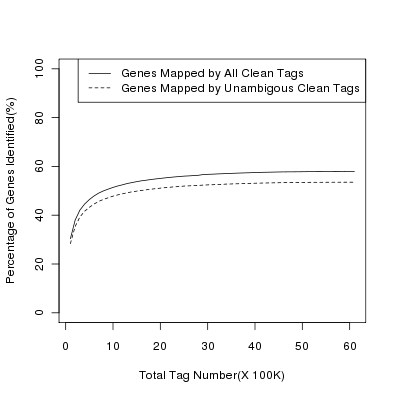 | 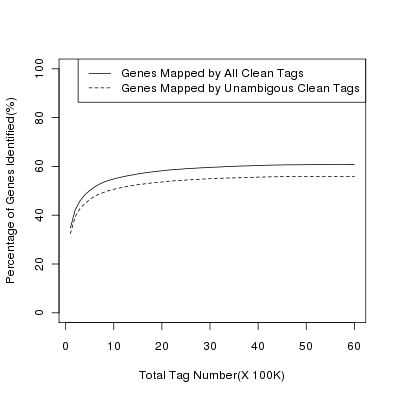 |

**Continues Table S1**

| Leaves-2 | Control | Salt stress |
| --- | --- | --- |
| 2 h | 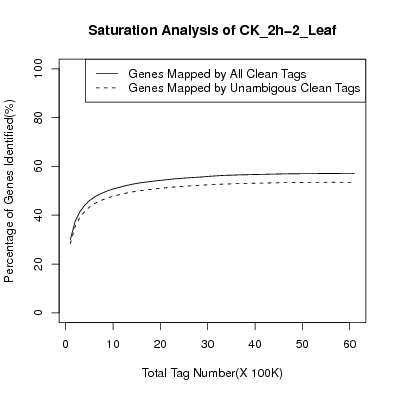 | 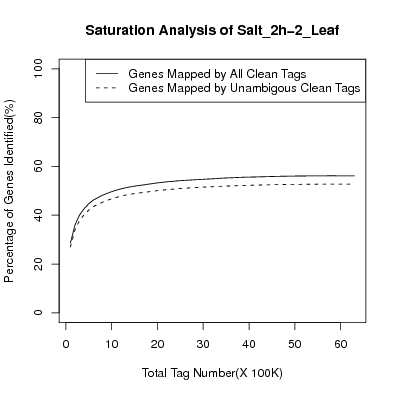 |
| 2 d | 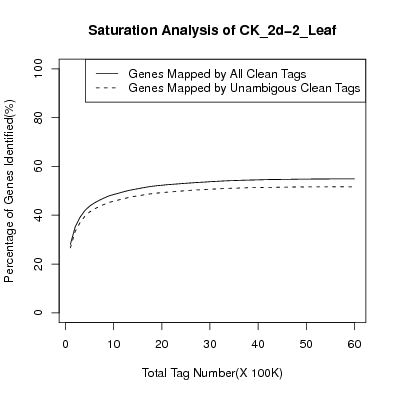 | 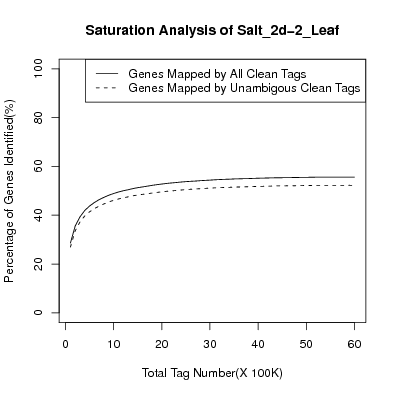 |
| 7 d | 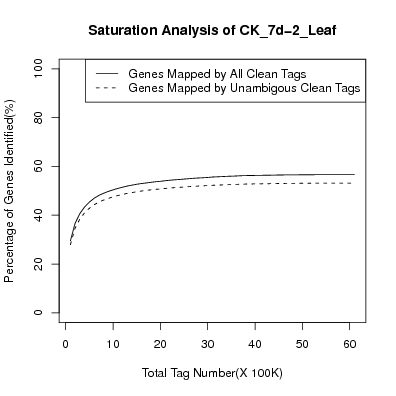 | 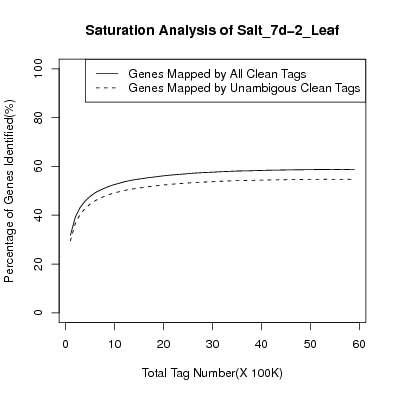 |
