# Supplementary material for: Global Analysis of Gene Expression Profiles in Physic Nut (Jatropha curcas L.) Seedlings Exposed to Salt Stress
Source: PLoS One. 2014 May 16;9(5):e97878. doi: 10.1371/journal.pone.0097878 (PMC4023963; doi:10.1371/journal.pone.0097878)
Supplement: Table S3 — Overview of salt-responsive genes in physic nut seedlings under salt stress. Fold change ≥1.8, p value<0.01 were counted. (DOCX) [file pone.0097878.s003.docx]

**Table S3.** **Overview of salt-responsive genes in physic nut seedlings under salt stress.**

Fold change ≥ 1.8, p value< 0.01 were counted.

| **Tissue** | **Gene number** | **Time after the onset of the salt stress** | | | **Total number** |
| --- | --- | --- | --- | --- | --- |
|  |  | **2 h** | **2 d** | **7 d** |  |
| **Roots** | Gene number in CK plants | 8283 | 8652 | 8375 | 9802 |
|  | Down-regulated genes (control/salt stress≥1.8) | 325 | 196 | 309 | 812 |
|  | Percentage (%) | 3.92 | 2.27 | 3.69 | 8.28 |
|  | Gene number in salt stress plants | 8646 | 8352 | 8339 | 9704 |
|  | Up-regulated genes (salt stress/control≥1.8) | 321 | 223 | 370 | 863 |
|  | Percentage (%) | 3.71 | 2.67 | 4.44 | 8.89 |
| **Leaves** |  | **2 h** | **2 d** | **7 d** |  |
|  | Gene number in CK plants | 5588 | 5148 | 4970 | 6301 |
|  | Down-regulated genes (control/salt stress≥1.8) | 109 | 89 | 111 | 299 |
|  | Percentage (%) | 1.95 | 1.73 | 2.23 | 4.96 |
|  | Gene number in salt stress plants | 5218 | 5198 | 5847 | 6711 |
|  | Up-regulated genes (salt stress/control≥1.8) | 23 | 91 | 722 | 826 |
|  | Percentage (%) | 0.44 | 1.75 | 12.35 | 12.31 |
